# Determination of a cost-effectiveness threshold for cancer interventions in Iran

**DOI:** 10.3389/fonc.2022.1039589

**Published:** 2022-12-12

**Authors:** Hossein Safari, Thomas G. Poder, Somayeh Afshari, Azin Nahvijou, Morteza Arab-Zozani, Nasrin Moradi, Hosein Ameri

**Affiliations:** ^1^ Health Promotion Research Centre, Iran University of Medical Sciences, Tehran, Iran; ^2^ Department of Management, Evaluation and Health Policy, School of Public Health, University of Montreal, Montreal, QC, Canada; ^3^ Centre de recherche de l’Institut universitaire en santé mentale de Montréal, CIUSSS de l’Est de l’île de Montréal, Montreal, QC, Canada; ^4^ Department of Health Management and Economics, School of Public Health, Tehran University of Medical Science, Tehran, Iran; ^5^ Cancer Research Center, Cancer Institute, Tehran University of Medical Sciences, Tehran, Iran; ^6^ Social Determinants of Health Research Center, Birjand University of Medical Sciences, Birjand, Iran; ^7^ Department of Health Management and Economics, School of Public Health, Iran University of Medical Science, Tehran, Iran; ^8^ Health Policy and Management Research Center, Department of Health Management and Economics, School of Public Health, Shahid Sadoughi University of Medical Sciences, Yazd, Iran

**Keywords:** cost-effectiveness threshold, quality-adjusted life-year, willingness to pay, cancer patients, Iran

## Abstract

**Background and objectives:**

The estimation of a cost- Effectiveness (CE) threshold from the perspective of those who have experienced a life-threatening disease can provide empirical evidence for health policy makers to make the best allocation decisions on limited resources. The aim of the current study was to empirically determine the CE threshold for cancer interventions from the perspective of cancer patients in Iran.

**Methods:**

A composite time trade-off (cTTO) task for deriving quality adjusted life-year (QALY) and a double-bounded dichotomous choice (DBDC) approach followed by open-ended question for examining patients’ willingness-to-pay were performed. A nationally representative sample of 580 cancer patients was recruited from the largest governmental cancer centers in Iran between June 2021 and January 2022, and data were gathered using face-to-face interviews. The CE threshold was calculated using the nonparametric Turnbull model and parametric interval-censored Weibull regression model. Furthermore, the factors that affect the CE threshold were determined using the parametric model.

**Results:**

The estimated CE threshold using the nonparametric Turnbull model and parametric interval-censored Weibull regression model was IRR 440,410,000 (USD 10,485.95) and IRR 595,280,000 (USD 14,173.33) per QALY, respectively. Gender, age, education, income, type of cancer, and current treatment status were significantly associated with the estimated CE threshold.

**Conclusions:**

The value of parametric model-based threshold in this study was 1.98 times the Iranian GDP per capita, which was lower than the CE threshold value recommended by the WHO (i.e., 3 times the GDP per capita) for low-and middle-income countries.

## Introduction

Iran is an ancient country located in the Middle East in which the public sector mainly provides primary, secondary, and tertiary health services. Iran’s health system has undergone several reforms during the past three decades, such as the establishment of National Health Network, the Family Physician Programme, integration of health services and medical education, and recently the Health Sector Evolution Plan (HSEP) (1). One of the main objectives of HSEP was to reduce health expenditure and to use the most effective interventions for patients. This target along with the introduction of technologies in Iran’s health system has led to decision making on health care resource allocation becoming increasingly important for policy makers (2). Decisions on resource allocation of healthcare interventions are usually made upon economic evaluation approaches such as cost-effectiveness analysis (CEA) and cost-utility analysis (CUA) (3). The CUA evaluates healthcare interventions with respect to their incremental cost per their quality adjusted life-year (QALY) gained (4). QALY is a multidimensional outcome that combine length of life with quality-of-life measures, and it reflects the health-related quality of life (HRQoL) (5). Incremental cost per QALY is a common measure of incremental cost-effectiveness ratio (ICER) (6). To make a decision, the obtained ICER is often compared with a cost-effectiveness threshold (CE threshold) to determine whether an intervention is cost-effective or not. The CE threshold can be estimated using expert opinions in the field, human capital approach, and willingness to pay (WTP) (7). The WTP is the most common approach and determines the threshold based on the maximum willingness-to-pay (WTP) per QALY (WTP/QALY) (3).

The WTP/QALY value can be derived from the perspectives of general population or specific group like patients. The general public’s perspective may be more appropriate for calculating the WTP/QALY value because health financing is often achieved from the general public, who are mainly taxpayers and potential patients (8). From the perspective of the general public, a number of hypothetical health states are evaluated by a representative sample which has not experienced hypothetical health states and has not been directly affected by healthcare interventions. Whilst from the perspective of patients, they are actually in the health states depicted and directly affected by healthcare interventions. Hence, it may be better suited for the estimation of the CE threshold because patients are best informed about a specific health state (9–11). In this line, it is generally recommended to consider both perspectives (12).

Among studies that have used the patients’ perspective for estimating the WTP/QALY, there is little empirical evidence of patients’ perspective with a life-threatening disease like cancer (7). A recent systematic review showed that only one study conducted in the Kingdom of Saudi Arabia drawn the value of WTP/QALY from a representative sample of cancer patients (13). However, countries usually rely on the CE threshold obtained from general public’s perspective to evaluate the interventions of life-threatening diseases (7). Evidence has demonstrated that the CE threshold of life-threatening diseases is often higher than that of other health conditions, then the use of the general public-based CE threshold may lead to interventions that are not cost-effective (14, 15). As a result, patients would have limited access to these interventions. To overcome this limitation, some countries have increased their threshold for more sever health states. For example in the UK, the threshold has increased from £30,000 to £50,000/QALY for life-threatening diseases (16, 17) and from €10,000 to €80,000/QALY in the Netherlands (18). However, the estimation of CE threshold from the perspective of those who experienced a life-threatening disease can provide empirical evidence to support interventions in resource allocation process. Thereafter, it would be interesting to specifically examine the WTP per QALY value from the perspective of specific disease groups (17). Cancers are one of the life-threatening diseases where new cases have significantly increased in recent years from 18.1 in 2018 to 19.3 million in 2020 worldwide (+6.63%) (19). In Iran, the number of new cancer cases in both sexes is predicted to increase from 131 191 in 2020 to 160 000 in 2025 (+21.96%) (20). The increase in cancers and subsequent increase in the use of treatment interventions like surgery, chemotherapy, radiotherapy, and hormonal therapy impose economic burden on patients, family and health system. Hence, the choice of effective treatment alternatives with respect to their costs (i.e., cost-effectiveness analysis) can support patients and improves financing and insurance reimbursement policies. In this way, having a specific CE threshold that reflects the perspective of cancer patients is important. The main objective of the present study was to empirically determine a CE threshold for cancer interventions using the perspective of cancer patients in Iran.

## Materials and methods

### Study design and data collection

A total of 565 inpatients and outpatients’ cancer (303 and 262, respectively) whose disease was pathologically confirmed were recruited using a consecutive sampling method. They were recruited from surgery, chemotherapy, and radiotherapy wards in the three largest governmental cancer centers of the provinces of Tehran, Isfahan, and Fars between June 2021 and January 2022. The population of these provinces corresponds to more than 25% of the Iran’s population in 2017, and patients are admitted to the centers from all over the country.

Data were collected through face-to-face interviews in patients’ rooms during a single visit in compliance with the ethical standards of the national research committee (approval no. IR.IUMS.SPH.REC.1398.205). The inclusion criteria were as follows: patients with healthy cognitive status who were able to read and write, and gave an informed consent to participate in the study.

### Study design

Respondents were first asked to provide demographic information and to self-rate their health state using the EQ-5D-5L. The EQ-5D-5L is currently the most common HRQoL instrument and includes five dimensions of health: mobility, self-care, usual activities, pain/discomfort, and anxiety/depression. The interim value set for the EQ-5D-5L is available for Iranian population (21). Interview procedure was performed in two stages: QALY measure followed by WTP estimate. After introducing the objectives of the study, demographic and clinical data were collected from patients’ self-report and their medical records, respectively. QALY was measured using a composite TTO (cTTO). The cTTO is a hybrid approach of the conventional TTO for valuing health states better than dead (BTD) and lead time TTO for valuing health states worse than dead (WTD). In the cTTO task, the patient was first asked to choose between living x years in full health followed by death (“Alternative 1”) or t years in the patient’s own current health state (where x≤t) followed by death (“Alternative 2”). Then, x was varied until the patient was indifferent between the two alternatives. The value of h, U (h), was computed as x/t. If the patient preferred to choose zero years in full health rather than the current health (i.e., the WTD state), lead TTO was assessed. In parallel, the patient was asked to choose between living for x years in full health followed by death (“Alternative 1”) or L years of lead time in full health followed by t years in the current health status and death (“Alternative 2”). Next, x was again varied till the patient was indifferent between the two alternatives (22). The value of h, U (h), was computed as x-L/t, where x < L and L=t. In an attempt to avoid the estimate bias, t was equal to 5 and 10 years for cancer patients with and without metastasis, respectively (**Appendix A**).

In order to estimate the WTP value, the patient was asked to image a hypothetical new treatment that can cure the cancer immediately and fully return to full health. Nevertheless, the treatment was not covered by health insurance or government. If the patient bought the treatment, it would benefit immediately and fully recover to perfect health until he died after X months. If the patient did not buy the treatment, he/she would live with the patient’s own current health state for X months and then die. The number of months (X) was computed as $\frac{1}{1-\text{current utility}}$ (23).

Maximum value of WTP/QALY was derived using a double-bounded dichotomous choice approach (DBDC) followed by open-ended question (24). Starting bid amounts were varied at 20%, 40%, 80%, 120%, 160%, 200%, 240%, 280%, and 320% of the Iranian GDP per capita in 2019 (300,000,000 IRR) (25), and were randomly presented for patients to avoid starting point bias. The bid amount was changed based on the yes/no answer to the first bid value. The bid amount increased and decreased by one level respectively based on the answers of yes and no. If the answer to the first bid was “yes” and then to the next level was “no”, the maximum WTP was determined as the midpoint between the two bids and if both bids were accepted, it was derived using an open-ended question. If the answer was “no” to the first bid but yes to the second bid, the maximum WTP was determined as the midpoint of the first and second bids and if both bids were rejected, it was determined using an open-ended question, which should be lower than the second bid offered. Nevertheless, the patient who was not willing to pay even a small amount was asked to indicate his/her reason. We reminded income and expenses of the patient’s household when they were presented the maximum WTP and also asked them to consider that the whole cost of the treatment had to be paid out of pocket in one time within a year. **Appendix B** presents an example of a WTP question for the treatment when the patient’s utility value was 0.2.

### Statistical analysis

The nonparametric Turnbull model (26) and the parametric interval-censored regression model with Weibull distribution (27) were used to value the mean WTP/QALY. The Turnbull model is one of the most widely used nonparametric distribution-free method for contingent valuation to estimate WTP (28). Turnbull model estimates the mean WTP as a lower bound estimate, hence this was attractive to many researchers and policy makers because its results are more conservative (26, 29). The lower bound mean estimate of WTP is calculated by the following equation:


$$E_{LB}\left(WTP\right)=\sum_{J=0}^{M^{*}}t_{j}(F_{j+1}^{*}-F_{j}^{*})$$

Where *M* ^*^ is the total number of bids after pooling back, *t_j_* the bidding amount for price *j*, F_j_^*^ the rate of the response “no” to price *j* after pooling back.

The estimates of the nonparametric model are based on the lower bound of each bid interval. In parallel, point estimates such as a median, and covariate analysis of factors associated with WTP cannot be provided by this model. To overcome the problems, a parametric interval-censored Weibull regression model was used. In this model, it is assumed that the WTP distribution is a Weibull distribution with the shape parameter θ and the scale σ. The mean and median of WTP using this model are calculated with the following equations:


Mean WTP=σ.Γ(1/θ +1)

$$Median\;WTP=\sigma {\left[-ln\left(0.5\right)\right]}^{1/\theta }$$

The parametric model not only provided point estimates but also made it possible to handle covariate analysis of the factors. To address the factors affecting respondents’ WTP/QALY, all relevant demographic and clinical variables were tested against WTP in the interval regression model and the non-significant variables at the 5% level were removed using backward elimination regression. The WTP/QALY values were also calculated based on US dollars using the exchange rate provided by the Iran Central Bank at the time of analysis (July 2022: IRR 42,000 = USD 1) (25).

## Results

Out of 580 patients who completed the EQ-5D-5L, the utility values of 565 patients were derived using the cTTO task. Fifteen interviews were excluded from final analysis due to being incomplete. Patients’ characteristics are presented in **Table 1**. Mean age of patients and mean duration of cancer were 51.55 (SD ± 13.7) years and 14.93 (SD ± 20.61) months, respectively. The largest number of cancer patients was colorectal cancer (23.01%)

**Table 1:** Patients’ characteristics (N=565).

Characteristics	N = 565n (%)
**Gender**	
Male	276 (48.85)
Female	289 (51.15)
**Age group (years)**	
≤39	125 (22.12)
40-49	114 (20.18)
50-59	157 (27.79)
60-69	116 (20.53)
≥70	53 (9.38)
**Education**	
Illiterate^a^	98 (17.35)
Primary	119 (21.06)
Secondary	223 (39.47)
University degree	125 (22.12)
**Marital status**	
Single	43 (7.61)
Divorced or widow	31 (5.49)
Married	491 (86.90)
**Status in household**	
Head of the household	238 (42.12)
Spouse of the head	271 (47.96)
Son/daughter of the head of the household	46 (8.14)
Parent of the head of the household	10 (1.77)
**Employment status**	
Having no income (unemployed or housespouse)	253 (44.78)
Having income (employed, self-employed or retired)	312 (55.22)
**Monthly household income** (**IRR)**	
<30,000,000	217 (38.41)
30,000,000 – 50,000,000	177 (31.33)
50,000,000 – 70,000,000	114 (20.18)
>70,000,0000	57 (10.09)
**Type of cancer**	
Colorectal	130 (23.01)
Lung	108 (19.12)
Breast	103 (18.23)
Stomach	76 (13.45)
Others	148 (26.19)
**Current treatment status**	
Chemotherapy	375 (66.37)
Chemotherapy and surgery	107 (18.94)
Chemotherapy, radiotherapy, and surgery	83 (14.69)
**Duration of disease since diagnosis (months)**	
≤5	212 (37.52)
6-11	150 (26.55)
12-23	95 (16.81)
≥24	108 (19.12)

a : they were able to read and write.

**Figure 1:**
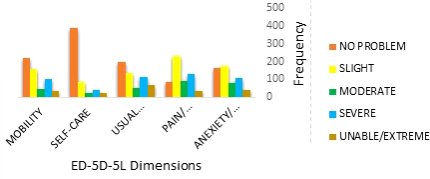
Distribution of problems reported on each EQ-5D-5L dimension.

**Figure 1** shows the distribution of problems reported by patients for each EQ-5D-5L dimension. As shown here, the patients reported the lowest frequency of problems for self-care followed by mobility, usual activity, anxiety/depression, and pain/discomfort dimensions. Patients reported “unable to/extreme problems” in all dimensions.

### WTP measures

The Turnbull estimates and the probability of answering “no” to each of the first bids for QALY gained are presented in **Table 2**. The response to the bid amounts of IRR 600,000,000; IRR 720,000,000; IRR 840,000,000; and IRR 960,000,000 are the ones that violate the monotonicity assumption for a standard distribution function. However, pooling the IRR 480,000,000; IRR 600,000,000; IRR 720,000,000; IRR 840,000,000; and IRR 960,000,000 responses ensured monotonicity. Probability mass point estimates are reported in columns F_j_* and f_j_*. Turnbull lower bound mean WTP/QALY was IRR 440,410,000 (SD 240,310,000) (USD 10,485.95) after excluding all zero WTP values.

**Table 2:** Turnbull estimates with pooling for WTP per QALY (N=565).

b_j_ (IRR)	Unrestricted estimates			Turnbull estimates			
	N_j_	T_j_	F_j_	N_j_*	T_j_*	F_j_*	f_j_*
60,000,000	1.000	38.000	0.026	1.000	38.000	0.026	0.026
120,000,000	3.000	67.000	0.045	3.000	67.000	0.045	0.018
240,000,000	6.000	83.000	0.072	6.000	83.000	0.072	0.028
360,000,000	8.000	89.000	0.090	8.000	89.000	0.090	0.018
480,000,000	13.000	81.000	0.160	38.000	288.000	0.132	0.042
600,000,000	13.000	77.000	0.169	Pooled	Pooled	Pooled	Pooled
720,000,000	6.000	33.000	0.182	Pooled	Pooled	Pooled	Pooled
840,000,000	4.000	36.000	0.111	Pooled	Pooled	Pooled	Pooled
960,000,000	2.000	61.000	0.033	Pooled	Pooled	Pooled	Pooled
288,000,000+	–	–	1.000	–	–	1.000	0.868

IRR, Rial; b_j_, bidding amount for price j; N_j_, the number of “no” responses to price j; T_j_, the number of observations in each bid, T_j_*, the number of observations after pooling back; F_j_*, probability of “no” response rate to price j after pooling back; f_j_* is the change of “no” response rate to price j after pooling back.

The results of the estimates obtained from the interval-censored Weibull regression model are presented in **Table 3**. The mean WTP/QALY value obtained from this model was IRR 595,280,000 (SD 311,610,000) (USD 14,173.33). The backward elimination analysis demonstrated that gender, age, education, employment status, household income, type of cancer, and current treatment status had a significant impact on patients’ WTP at the level of 5%, so they remained in the parametric regression model. The effect of all variables on WTP was positive except for age, gender, and treatment status (**Table 3**).

**Table 3:** Results of Weibull regression analysis for WTP per QALY (*n* = 565).

Variable	Estimate	SE	Z value	P value	[95% Conf. Interval]
**Female (male = ref.)**	-0.232	0.109	-2.12	0.034	[-0.447 -0.017]
**Age (y)**	-0.005	0.003	-2.00	0.043	[-0.015 0.006]
**Education (illiterate = ref.)**					
Primary	0.078	0.101	0.77	0.439	[-0.120 0.278]
Secondary	0.182	0.099	1.84	0.065	[-0.011 0.377]
University	0.276	0.122	2.26	0.024	[0.036 0.515]
**Having income (Having no income = ref.)**	0.229	0.108	2.13	0.034	[0.017 0.441]
**Monthly household income (<30 = ref.)**					
30-50	0.337	0.078	4.31	0.000	[0.183 0.490]
50-70	0.768	0.095	8.05	0.000	[0.581 0.955]
70<	0.971	0.129	7.49	0.000	[0.716 1.22]
**Type of cancer (colorectal = ref.)**					
Lung	0.376	0.105	3.55	0.000	[0.168 0.583]
Breast	0.065	0.098	0.66	0.509	[-0.128 0.259]
Stomach	0.006	0.115	0.06	0.955	[-0.219 0.232]
Other	0.335	0.096	3.47	0.001	[0.145 0.525]
**Current treatment (Chemotherapy=ref.)**					
Chemotherapy and surgery	-0.028	0.089	-0.32	0.747	[-0.204 0.146]
Chemotherapy, radiotherapy, and surgery	-0.282	0.094	-3.00	0.003	[-0.467 -0.098]
**Constant**	3.685	0.201	18.31	0.000	[3.29 4.08]
**Log likelihood = -931.564**					

The acceptance rate to the first question has a specific pattern with respect to the characteristic of each variable. Indeed, this probability was higher for male, younger, educated, and richer patients, and for those who were employed and diagnosed with more severe cancers (lung and colorectal cancers) and were receiving more severe treatment (**Table 4**).

**Table 4:** Acceptance rate to the first question among significant variables.

Characteristic	N = 565 n (%)	Number of yes answers to the first question	Acceptance rating
**Gender**			
Male	276 (48.85)	250	0.91
Female	289 (51.15)	252	0.87
**Age group (years)**			
≤39	125 (22.12)	116	0.93
40-49	114 (20.18)	104	0.91
50-59	157 (27.79)	142	0.90
60-69	116 (20.53)	105	0.90
≥70	53 (9.38)	43	0.81
**Education**			
Illiterate ^a^	98 (17.35)	87	0.88
Primary	119 (21.06)	106	0.89
Secondary	223 (39.47)	203	0.91
University degree	125 (22.12)	115	0.92
**Employment status**			
Having no income (unemployed or housespouse)	253 (44.78)	225	0.89
Having income (employed, self-employed or retired)	312 (55.22)	284	0.91
**Monthly household income** (**IRR)**			
<30,000,000	217 (38.41)	195	0.90
30,000,000 – 50,000,000	177 (31.33)	154	0.87
50,000,000 – 70,000,000	114 (20.18)	106	0.93
>70,000,0000	57 (10.09)	54	0.95
**Type of cancer**			
Colorectal	130 (23.01)	119	0.91
Lung	108 (19.12)	101	0.93
Breast	103 (18.23)	90	0.87
Stomach	76 (13.45)	67	0.88
Others	148 (26.19)	132	0.89
**Current treatment status**			
Chemotherapy	375 (66.37)	339	0.90
Chemotherapy and surgery	107 (18.94)	94	0.88
Chemotherapy, radiotherapy, and surgery	83 (14.69)	71	0.85

a : they were able to read and write.

## Discussion

The aim of this study was to calculate the mean value of WTP per QALY from the perspective of cancer patients in Iran. The mean WTP/QALY value was derived from a nationally representative sample of cancer patients using the nonparametric Turnbull model and the parametric interval-censored Weibull regression model. The sample is considered to be representative of cancer patients in Iran since they were selected from the three largest cancer centers receiving patients from all over Iran, in addition to present different socioeconomic status. The estimated CE threshold estimated using Turnbull model and Weibull regression model was IRR 440,410,000 (USD 10,485.95) and IRR 595,280,000 (USD 14,173.33) per QALY, respectively. The impacts of gender, age, education, income, type of cancer, and current treatment status on the estimated CE threshold were statistically significant.

The distribution of problems reported by patients on each EQ-5D-5L dimension showed that the lowest frequency of problems was for self-care followed by mobility, usual activity, anxiety/depression, and pain/discomfort dimensions. The distribution was similar to what was reported in other studies conducted on cancer patients in Iran (30–32).

The DBDC approach followed by open-ended question was used to examine patients’ WTP because it produces a more accurate set of WTP values and familiarizes respondents with the yes or no pricing questions, and gives more freedom to participants to express the value that might not be present within the pre-determined values in a DBDC approach (33, 34). Furthermore, this approach reduces the strategic bias in participants compared to the open-ended method alone (35, 36) and, compared to single double-bound dichotomous choice format, it provides more efficient estimates of central tendency (36).

The CE threshold was calculated using the nonparametric Turnbull model and parametric interval-censored Weibull regression model. The interval-censored responses generated from the DBDC and open-ended questions were analyzed using the parametric interval regression models (37, 38). The models allowed us to estimate the effects of factors associated with respondents’ WTP and provided point estimates such as median. Nevertheless, the models rely on *a priori* assumptions about the underlying distribution function of respondents’ WTP. Hence, violation of assumptions may result in inaccurate estimates of parameters (38). An appropriate alternative to parametric estimation is the use of distribution-free methods. The preferred distribution-free estimation method was the nonparametric estimation proposed by Turnbull. It is also useful for the responses of dichotomous or categorical variables and provides the most conservative estimates of WTP (26, 29), thus limiting the hypothetical bias. The value of CE threshold calculated using the nonparametric and parametric models was IRR 440,410,000 and IRR 595,280,000, respectively. The results obtained from interval-censored data using the nonparametric and parametric models cannot be directly compared because the nonparametric Turnbull model provides a probability only for left censored observations (39). The overall performance of the parametric model in interval-censored data appears to be highly satisfactory, especially when the Weibull distribution is applied, because it permits a wide range of distributional shapes to be fitted (39). On the other hand, the parametric method allows the inclusion of covariates in the modeling of WTP estimates. Thus, the result of parametric model can be more useful for the discussion in this study. Moreover, this did not imply that the nonparametric method is inferior to the parametric method. The result of nonparametric Turnbull method could then be used as a reference (23).

The value of parametric model-based CE threshold in this study was 1.98 times the Iranian GDP per capita. This was lower than the threshold value of 3 times the GDP per capita recommended by the WHO for low-and middle-income countries (40), while the range of first bids put forward in this research was between 0.2 and 3.2 of the GDP per capita in Iran. The estimated CE threshold value (USD 14,173) was greater than the highest monetary value of a QALY obtained from two studies conducted on patients with diabetes (USD 5043) (41)and cardiovascular disease (USD 3599) (14). Also, our result compared to two studies conducted by Lankarani et al. (USD 2847) (42) and Moradi et al. (USD 2666) (43) on the general public in Iran. The greater WTP in patients compared with general population was reported by a recent systematic review (7). The high value of WTP per QALY in our sample is supported by the high value of WTP/QALY for cervical cancer in Taiwan (USD 21,221.96) (44). These comparisons indicate that countries should change the threshold based on the severity of health states, as some countries such as the UK and the Netherlands increased the threshold for life-threatening diseases (17, 18). When comparing our results (1.98 times GDP per capita) with the WHO CE threshold value, it is likely to raise a major issue in using the WHO CE threshold in the country, because it may result in reimbursing many health care interventions that are not efficient with respect to costs and effectiveness. The low local estimated CE threshold value compared to the WHO threshold was reported in other studies in Iran (42) and other middle-income countries such as Thailand (45) and Malaysia (23). Overall, the findings indicated that the CE threshold recommended by the WHO should be employed with considerable caution in making decisions and allocating resources to cost-effective interventions.

Including covariates in interval-censored Weibull model revealed that gender, age, education, employment status, household income, type of cancer, and current treatment status were significant predictors of patients’ WTP. The increase of income and age has respectively significant positive and negative influence on the values of WTP/QALY. These findings were found in most of the studies conducted in middle-income countries (23, 41–44, 46) and are supported by the recent systematic review (7). It demonstrated that a 1% increase in income is associated with a 0.6% increase in the WTP value, and a 1-year increase in age is associated with a 3.3% decrease in the WTP value (7). Compared to male patients, female patients tended to have a lower WTP, which was also observed in previous studies (7), independently of income. The current treatment status of patients was another factor with a significant negative influence on the WTP value. Patients receiving more severe treatment were less willing to pay for the hypothetical treatment described in the questionnaire. This finding may indicate that patients receiving more severe treatment did not have a good experience of treatments. Employed patients and those with a higher level of education reported higher levels of WTP/QALY. These findings were similar to studies in Malaysia (23), Thailand (47), and two other studies in Iran (42, 43), while it was not supported by the results of the recent systematic study (7).

One limitation that should be noted is that our sample may not be perfectly representative of the cancer patients, even though they were recruited from three largest cancer centers in three major provinces of Iran with more than 25% of Iran’s population that admitted patients from all over the country.

## Conclusion

The estimated CE threshold in this study for cancer patients was IRR 595,280,000 (USD 14,173), which was lower than the currently used WHO-recommended threshold value (1.98 versus 3 times the Iranian GDP per capita). Gender, age, education, employment status, monthly household income, type of cancer, and current treatment status were factors that significantly affected the value of the determined CE threshold.

## Data availability statement

The original contributions presented in the study are included in the article/**Supplementary Material**. Further inquiries can be directed to the corresponding author.

## Ethics statement

All procedures performed in studies involving human participants were in accordance with the ethical standards of the national research committee (approval no. IR. IUMS.1400.398) in Iran. The patients/participants provided their written informed consent to participate in this study.

## Author contributions

Study design and statistical analysis and interpretation of thedata: HS, HA, and NM; drafting of the manuscript: HA, SA, andMA-Z; critical revision of the manuscript for importantintellectual content: HA, TGP, and AN. All authors contributedto the article and approved the submitted version.
