# Supplementary material for: Determination of a cost-effectiveness threshold for cancer interventions in Iran
Source: Front Oncol. 2022 Dec 12;12:1039589. doi: 10.3389/fonc.2022.1039589 (PMC9791211; doi:10.3389/fonc.2022.1039589)
Supplement: Supplementary file 1 [file DataSheet_1.docx]

**Appendix A**

**Example of composite time trade-off task with 5 years survival**

**A-1. Health States Better than Dead**

Please image there are Life A and Life B for you. Life A shows that you could live 5 years in full health and then die immediately, and Life B indicates you could live in your own current health state for 5 years and then die immediately. Now, the number of years in Life A decreases but the number of years with your own current will remain unchanged, kindly tell me in which year of Life A it doesn't matter to you whether you live in Life A or Life B.

Which life will you choose?

| Life A | 5 years in the full health | Death |
| --- | --- | --- |
|  |  |  |
| Life B | 5 years in the current health | Death |

**A-2. Health States Worse than Dead**

Please image there are Life A and Life B for you. Life A shows that you could live 5 years in full health and then die immediately, and Life B indicates you could live 5 years in full health years followed by 5 years in the current health state and then die immediately. Now, the number of years in Life A decreases but the number of years in Life B will remain unchanged, kindly tell me in which year of Life A it doesn't matter to you whether you live in Life A or Life B.

Which life will you choose?

| Life A | 5 years in the full health | | Death |
| --- | --- | --- | --- |
|  |  | |  |
| Life B | 5 years in the full health | 5 years in the current health | Death |
