# Supplementary material for: Determination of a cost-effectiveness threshold for cancer interventions in Iran
Source: Front Oncol. 2022 Dec 12;12:1039589. doi: 10.3389/fonc.2022.1039589 (PMC9791211; doi:10.3389/fonc.2022.1039589)
Supplement: Supplementary file 2 [file DataSheet_2.docx]

**Example of a Willingness to Pay Question for Utility=0.2**

| Please image you will die after 1 year and 3 months. Now, assume that there is a new treatment without pain and no adverse effect that can make you immediately and fully recover to perfect health. However, the treatment is not covered by health insurance or government, so you will have to pay out of pocket for the whole cost of the treatment in one time within a year. If you do not buy the treatment, you would live with your own current health state for 1 year and 3 months and then die. | |
| --- | --- |
| Please consider income and expenses of your household before making your decision. You can borrow money, but have to pay out of pocket the whole cost of the treatment in one time within a year.  120,000,000 IRR | |
| 1.Would you be willing to pay ……………………. for the treatment? (e.g., First bid=120,000,000 IRR) | |
| 1. Yes 🡪Go to Question 2 | 1. No 🡪Go to Question 4 |
|  |  |
| 2.Would you be willing to pay.……………………...for the treatment?  240,000,000 IRR | |
| 1. Yes 🡪Go to Question 3 | 1. No |
|  |  |
| **3. What is the maximum amount you are willing to pay for the treatment?** | |
|  |  |
| 4. Would you be willing to pay ……………………. for the treatment?  60,000,000 IRR | |
| 1. Yes | 1. No 🡪Go to Question 5 |
|  |  |
| **5. What is the maximum amount you are willing to pay for the treatment?**  **(**This amount must be lower than 60,000,000 IRR**)** | |
